# Association between maternal pregnancy complications and long-term depressive and anxiety disorders: evidence from the UK millennium cohort study

**DOI:** 10.1007/s00127-026-03094-4

**Published:** 2026-04-29

**Authors:** Elizabeth O. Bodunde, Fergus P. Mccarthy, Karen O’Connor, Karen Matvienko-Sikar, Ali S. Khashan

**Affiliations:** 1 School of Public Health, University College Cork, Western Gateway Building, Cork, Ireland; 2 INFANT Research Centre, University College Cork, Cork, Ireland; 3 Department of Obstetrics and Gynaecology, Cork University Maternity Hospital, Cork, Ireland; 4 Early Intervention in Psychosis Team, RISE, South Lee Mental Health Services, Cork, Ireland; 5 Department of Psychiatry and Neurobehavioral Science, University College Cork, Cork, Ireland

**Keywords:** Pregnancy, Depression, Anxiety, Epidemiology, Maternal health

## Abstract

**Purpose:**

Limited evidence exists on the association between maternal pregnancy complications and depressive and/or anxiety disorders in the longer term. We examined the association between pregnancy complications and later depressive and anxiety disorders.

**Methods:**

The study cohort consisted of mothers who participated in the UK Millennium Cohort Study. Pregnancy complications were self-reported at 9 months postpartum and categorised into 0, 1, 2, and 3 + complications. Follow-up surveys, including questions about clinically diagnosed depressive, and anxiety disorders were carried out at 3, 5,7,11, and 14 years postpartum. The primary outcome is the diagnosis of depressive and anxiety disorders, in the mother up to 14 years postpartum. We applied multivariable logistic regression models adjusting for several potential confounders.

**Results:**

10,510 mothers were included in the analyses, with *n* = 3935 (37%) of mothers experiencing at least one pregnancy complication. The fully adjusted odd ratios (aOR) were, [aOR, 1.54 (95% CI, 1.40–1.70)], [aOR, 1.70 (95% CI, 1.46–1.99)], [aOR, 2.11 (95% CI, 1.65–2.69)] for 1, 2, and 3 + pregnancy complications respectively. Bleeding in later pregnancy [OR, 1.33 (95% CI,1.08–1.65)], preeclampsia [OR, 1.43 (95% CI, 1.22–1.67)], threatened miscarriage [OR, 1.38 (95% CI, 1.16–1.64)], were associated with later depressive and anxiety disorders. No association was observed for gestational diabetes mellitus [OR, 1.01 (95% CI, 0.74–1.37)].

**Conclusion:**

An increasing number of pregnancy complications was associated with higher odds of depressive and anxiety disorders up to 14 years postpartum. Efforts to minimise and manage multiple pregnancy complications may benefit women’s long-term mental health.

**Supplementary Information:**

The online version contains supplementary material available at https://doi.org/10.1007/s00127-026-03094-4.

## Introduction

The World Health Organisation (WHO) estimates that as many as 15% of pregnancies will develop potentially life-threatening complications [1]. Some pregnancy complications may resolve at childbirth or shortly thereafter, while other pregnancy complications have been associated with longer-term adverse health problems [2, 3]. For instance, there are described associations between pregnancy complications and increased risks of cardiovascular [4], later life-renal disorders [5], and type 2 diabetes mellitus [6]. Beyond physical health problems, there is established evidence that pregnancy complications may be a risk factor for developing mental health disorders [3]. An extensive systematic review and an umbrella review found the risk of postpartum depression and postpartum anxiety to be increased among mothers who have experienced complications [7]. However, most studies have focused on the short-term postpartum [8–10]; there has been less focus on the medium to longer-term risks of a woman developing depressive and anxiety disorder.

Inconsistent associations have been observed between pregnancy complications and longer-term depressive and anxiety disorders, and studies have focused on pregnancy losses [11]. Some studies have reported the risk of depressive and anxiety disorders beyond 12 months postpartum to be two to three times increased in women with miscarriage and terminated pregnancies [12, 13], but this has been contravened in other studies [14, 15]. A few studies found preeclampsia [16, 17] and gestational diabetes mellitus [18] to be associated with later-life depressive and anxiety disorders. However, other studies reported no associations [19–21]. Similarly, the link between threatened miscarriage and bleeding in later pregnancy has focused on depressive and anxiety disorders in the immediate postpartum period [22–24].

Despite the large body of evidence on maternal mental health during the postpartum period, available evidence on long-term mental health disorders is limited. Recent studies report that women may be at increased risk of mental health disorders not only immediately after pregnancy and childbirth but also beyond the postnatal period [25–27]. For instance, a systematic review examining maternal consequences of postpartum depression suggest that women who experienced postnatal mental disorders may continue to be at risk for subsequent mental disorders up to 3 years postpartum [25]. Furthermore, a large cohort study using data from the UK biobank found that incident cases of mood disorders including depression, anxiety and stress-related disorders were significantly elevated for women with a history of stillbirth [Hazard ratio (HR; 1.21, 95% CI, 1.08–1.19), miscarriage (HR 1.14, 95% CI, 1.08–1.19) and termination of pregnancy (HR 1.39, 95% CI, 1.30–1.48) during a median follow-up of 13.36 years [26]. However, existing literature have focused on pregnancy loss, with comparatively fewer research on other pregnancy complications.

The extent to which observed associations between pregnancy complications is linked with subsequent mental health disorders remains unclear, beyond pregnancy losses. Other pregnancy complications such as preeclampsia, threatened miscarriage, gestational diabetes mellitus, and bleeding during pregnancy are among the most prevalent obstetric complications encountered in clinical practice [28]. Therefore, this present study explored the association between pregnancy complications and maternal depressive and anxiety disorders across 14 years postpartum using the nationally representative UK Millennium Cohort Study. We aimed to investigate (1) the association between cumulative pregnancy complications and depressive and anxiety disorders in the 14 years postpartum; (2) whether preeclampsia, threatened miscarriage, bleeding in later pregnancy, and GDM were associated with depressive and anxiety disorders in the 14 years postpartum.

## Methods

### Design and study sample

**Fig. 1:**
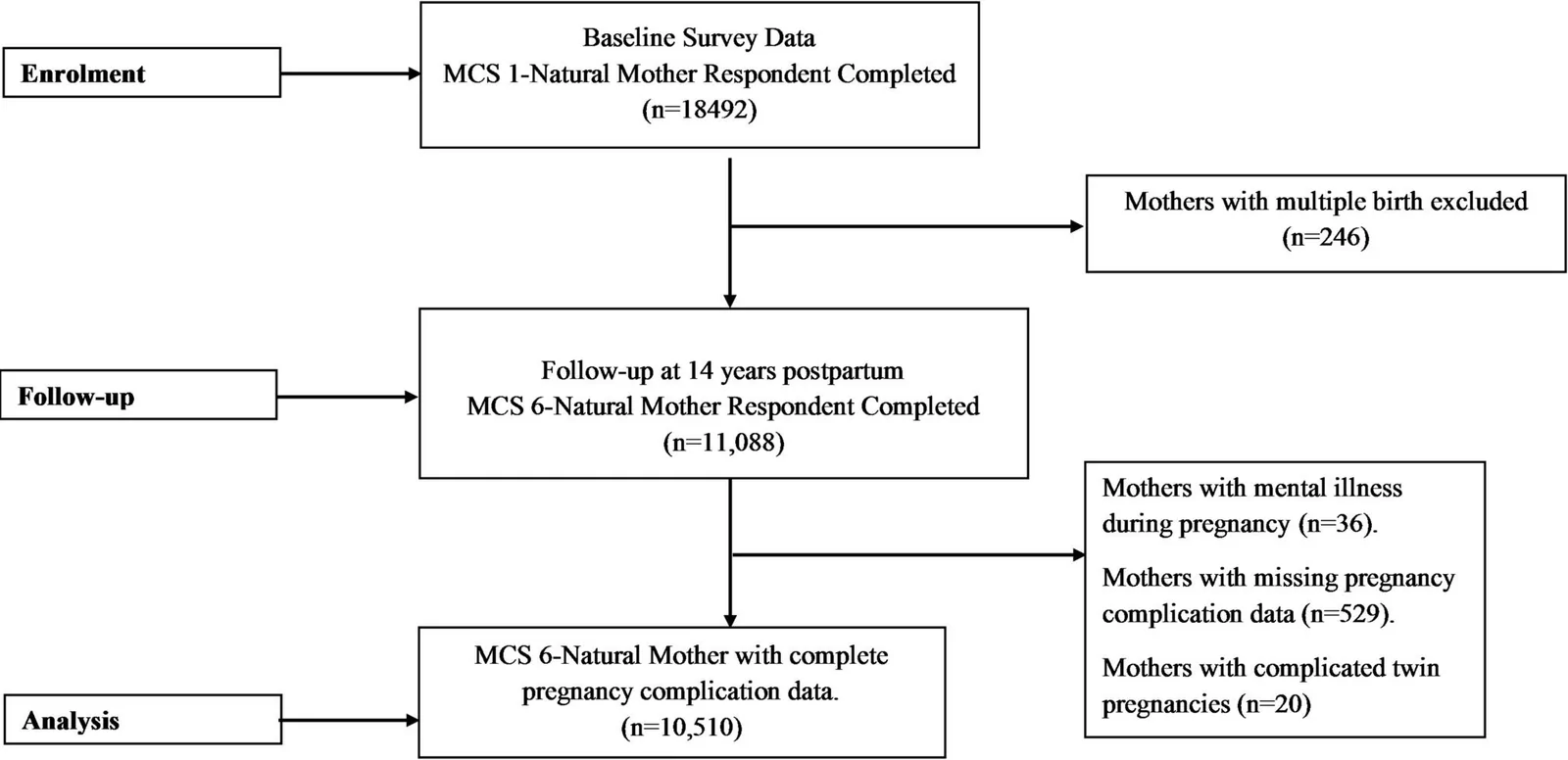
Flow diagram of sample determination

We performed a secondary data analysis of the UK Millennium Cohort Study (MCS) following the Strengthening the Reporting of Observational Cohort Studies in Epidemiology (STROBE) guidelines [29], see appendix S1. The MCS is a nationally representative prospective cohort study that included 18,818 children born in the UK between 2000 and 2002. The details of the study design and data collection have been described elsewhere in detail [30]. In summary, all live births in England, Scotland, Wales, and Northern Ireland were sampled at 9 months using stratified sampling by electoral wards. Survey interviews were conducted with biological mothers (*n* = 18,492) recruited to the cohort at 9 months postpartum (MCS1). There were six subsequent follow-ups at 3 years postpartum (MCS2), 5 years postpartum (MCS3), 7 years postpartum (MCS4), 11 years postpartum (MCS5), 14 years postpartum (MCS6), and 17 years postpartum (MCS7). As attrition is a problem common to most cohort studies, [31] the number of mothers by 14 years postpartum had declined to 11,088 (60% of the sample at 9 months postpartum). We excluded mothers at 17 years postpartum as the data available focused on the cohort member (child) and not the mother. We restricted this current study to mother-child pairs with data available at any time up to 14 years postpartum. Mothers with multiple births, mental illness in pregnancy, and missing pregnancy complication data were excluded, see Fig. 1.

### Exposures

In the MCS, mothers were asked at the 9-month interviews, ‘Did you have any problems or illnesses during pregnancy that required medical treatment?’ If they responded positively, they were asked ‘What illnesses or problems did you have?’ see appendix S2. For the primary exposure, we counted the total number of pregnancy complications for each mother and categorised them into 1, 2, and 3 or more pregnancy complications. Preeclampsia, threatened miscarriage, bleeding in later pregnancy, and GDM were considered secondary exposures, and each complication was analysed individually [32]. If mothers responded negatively to the initial question of whether they had experienced any problem or illness during pregnancy, we considered “no pregnancy complication”.

### Outcomes

Data on depressive/anxiety disorders were collected as a single composite variable. Mothers were asked at 9 months postpartum, and at all subsequent follow-ups up to 14 years postpartum if they had ever been told by a doctor that they were suffering from depression or severe anxiety. The primary outcome in this study was a cumulative diagnosis of depression/anxiety (ever diagnosed with depression or severe anxiety) across the 14 years postpartum. This was based on whether the mother reported a diagnosis of depression or severe anxiety at any time point from 9 months up to 14 years postpartum. We considered cumulative diagnoses at each time points (at 9 months, 3,5,7,and 11 years postpartum) as secondary outcomes.

### Covariates

Because of their potential relationship with both pregnancy complications and depressive and anxiety disorders [33], we considered the following potential confounders in the analysis: Maternal age at the time delivery (14−19 years, 20–29 years, 30–39 years, 40 + years), maternal BMI calculated as weight in kilograms divided by height in meter squared (underweight, normal weight, overweight, and obese), highest educational level attained (none, less than O–level, O–level, A–level, diploma/higher), maternal ethnicity (White, Others), parity (multiparous, primiparous), smoking status during pregnancy (non-smoker, quit smoking, smoking during pregnancy). Mothers were asked if they had a longstanding illness, and diagnoses were grouped by ICD-10 code [32]. This information was categorised as a dichotomous variable (yes, no). Other variables included household income based on the Organisation for Economic Co-operation and Development (OECD) equalized income quintiles. Area deprivation was based on indices of multiple deprivation grouped by the most deprived to the least deprived deciles. We also included infant variables, including child sex (male, female).

Postpartum psychological distress was measured at baseline (9 months postpartum) using the modified nine-item Rutter Malaise Inventory (RMI-9) with an overall RMI score ≥ 4, indicating a sign of psychological distress [34]. Mothers were asked if their child was admitted to a special baby unit after birth (yes, no). Preterm birth is calculated by the child’s gestation age in days and converted to ≥ 37 weeks gestation (yes, no). In two separate questions, mothers were asked at 7 years postpartum if their child had been clinically diagnosed with autism or attention-deficit hyperactivity disorders (yes, no). The description of covariates is provided in appendix S3.

### Statistical analysis

We compared the distribution of participants’ characteristics in relation to pregnancy complications using Ӽ^2^ statistics: maternal age, maternal pre-pregnancy BMI, maternal ethnicity, area deprivation, alcohol consumption in pregnancy, maternal smoking during pregnancy, maternal educational level, parity, longstanding illnesses, and child sex.

To examine the association between pregnancy complications and cumulative depressive and anxiety disorders by 14 years postpartum, four logistic regression models were conducted. Logistic regression was used to estimate the crude and adjusted odds ratios (aOR), and 95% confidence intervals (CI) for the likelihood of depressive and anxiety disorders among mothers who had pregnancy complications compared to those who did not. Model 1 is the crude model. Model 2 was adjusted for maternal age, ethnicity, area deprivation, education level, and household income. Model 3 was adjusted for maternal BMI, maternal smoking, alcohol consumption in pregnancy, longstanding illnesses, parity, and child’s sex. Model 4 included all covariates in models 2 and 3. This allowed us to evaluate both the crude and the extent to which the associations are explained by relevant potential confounders. In a separate analysis, we assessed the association between specific pregnancy complications (preeclampsia, threatened miscarriage, bleeding in later pregnancy, and GDM) and depressive and anxiety disorders by 14 years postpartum using a crude (Model 1) and fully adjusted model (Model 2).

Subgroups and sensitivity analyses were conducted to determine whether the association between pregnancy complications and depressive and anxiety disorders by 14 years postpartum was modified by key maternal and child characteristics. Effect modification was assessed by the presence of a longstanding illness (yes, no), parity (primiparous, multiparous), area deprivation (least deprived, most deprived), child sex (male, female), and in a posthoc analysis, ethnicity (white, others) as these factors may influence both pregnancy complications and long-term depressive and anxiety disorders. To further explore potential effect modifications, we fitted interaction terms between pregnancy complications and (1) postnatal psychological distress (at 9 months postpartum), (2) preterm birth, (3), NICU admission, and (4) child’s neurodevelopmental disorders by age 7, and in a posthoc analysis (5) support after birth.

We conducted sensitivity analyses to assess the likelihood of potential bias arising from missing data and sample attrition over the follow-up period. Specifically, in a complete-case sensitivity analysis, we examined the associations between pregnancy complications and depressive and anxiety disorders, among participants with complete exposure and confounder data at all time points up to 14 years postpartum. Finally, we repeated the primary analysis among mothers who participated in wave 6 (14 years postpartum) only. Missing data on potential confounders were handled using a missing category. All statistical tests used a significance level of *P* < 0.05. All analyses were conducted using Stata, version 16.0 (Stata Corp).

## Results

### Participant Characteristics

A total of 10,510 biological singleton mother-child pairs were included in the study. The flow diagram of participants is shown in Fig. 1. Whilst we considered four pregnancy complications (threatened miscarriage, bleeding in later pregnancy, preeclampsia, and GDM), the primary exposure of interest was the total number of pregnancy complications. 6575 (63%) of mothers experienced no pregnancy complications while 3935 (37%) of mothers had at least one complication during pregnancy. The age of the mothers ranged from 14 to 48 years with a mean ± sd of 29.0 ± 5.7 years. Compared to mothers without pregnancy complications, mothers with pregnancy complications were more likely to have higher BMI (24.2 ± 4.7 kg/m^2^), to have a male child (51.3%), and to be first-time mothers (52.3%). Overall, across 14 years postpartum, 47.8% of mothers had reported at least one diagnosis of depressive and anxiety disorders. The sociodemographic characteristics of the mothers are presented in Table 1.

**Table 1:** Demographic characteristics of study participants

Characteristics, *n* (%)	Pregnancy Complications		Total 10,510 (100)
	^a^No 6575 (62.6)	Yes 3935 (37.4)	
Maternal age			
*Mean (SD)*	*29.0 (5.7)*	*28.9 (5.8)*	*29.0 (5.7)*
14–19	408 (6.2)	256 (6.5)	664 (6.3)
20–29	2910 (44.3)	1771 (45.0)	4681 (44.5)
30–39	3094 (47.1)	1824 (46.4)	4918 (46.8)
40+	161 (2.5)	84 (2.1)	245 (2.3)
Missing	2 (0.0)	-	2 (0.0)
^b^Maternal BMI			
*Mean (SD)*	*23.5 (4.2)*	*24.2 (4.7)*	*23.8 (4.4)*
Normal (18.5–24.9 kg/m^2^)	4064 (61.8)	2240 (56.9)	6304 (60.0)
Underweight (< 18.5 kg/m^2^)	315 (4.8)	186 (4.7)	501 (4.8)
Overweight (25–29.9 kg/m^2^)	1182 (18.0)	816 (20.8)	1998 (19.0)
Obese (30–39 kg/m^2^)	463 (7.0)	402 (10.2)	866 (8.2)
Missing	551 (8.4)	291 (7.4)	842 (8.0)
Child sex			
Female	3266 (49.7)	1915 (48.7)	5188 (49.3)
Male	3308 (50.3)	2019 (51.3)	5340 (50.7)
Missing	1 (0.0)	1 (0.0)	2 (0.0)
^b^Ethnicity			
White	5417 (82.4)	3400 (86.4)	8817 (83.9)
Non-white	1146 (17.4)	530 (13.5)	1676 (15.9)
Missing	12 (0.2)	5 (0.1)	17 (0.2)
^b^Maternal Education			
None of these	1094 (16.6)	529 (13.4)	1623 (15.4)
> O level	634 (9.6)	389 (9.9)	1022 (9.7)
O level	2052 (31.2)	1368 (34.8)	3420 (32.5)
A level	649 (9.9)	403 (10.2)	1052 (10.0)
Diploma/Higher	1943 (29.6)	1137 (28.9)	3080 (29.5)
Other qualification	195 (3.0)	103 (2.6)	298 (2.8)
Missing	8 (0.1)	6 (2.0)	14 (0.1)
^b^Area deprivation			
Most deprived	1955 (29.7)	1091 (27.7)	3046 (29.0)
Second decile	1435 (21.8)	889 (22.6)	2324 (22.1)
Third decile	1155 (17.6)	689 (17.5)	1844 (17.6)
Fourth decile	938 (14.3)	633 (16.1)	1571 (14.9)
Least deprived	1092 (16.6)	633 (16.1)	1725 (16.4)
Longstanding illness			
No	5558 (84.5)	2738 (69.6)	8295 (78.9)
Yes	1014 (15.4)	1195 (30.3)	2209 (21.0)
Missing	3 (0.1)	2 (0.1)	5 (0.1)
^b^Parity			
Multiparous	3342 (50.8)	1877 (47.7)	5219 (49.7)
Primiparous	3233 (49.2)	2058 (52.3)	5291 (50.3)
^b^Household income			
Lowest quintile	1372 (20.9)	763 (19.4)	2135 (20.3)
Second quintile	1343 (20.4)	830 (21.1)	2173 (20.7)
Third quintile	1240 (18.9)	800 (20.3)	2040 (19.4)
Fourth quintile	1311 (19.9)	814 (20.7)	2125 (20.2)
Highest quintile	1292 (19.6)	724 (18.4)	2016 (19.2)
Missing	17 (0.3)	4 (0.1)	21 (0.2)
^b^Maternal smoking in pregnancy			
Nonsmoker	4984 (75.8)	2857 (72.6)	7838 (74.6)
Quit smoking	707 (10.8)	516 (13.1)	1223 (11.6)
Smoked during pregnancy	887 (13.4)	562 (14.3)	1449 (13.8)
^b^Alcohol consumption in pregnancy			
No	4528 (68.9)	2778 (70.5)	7306 (69.5)
Yes	2044 (31.0)	1154 (29.4)	3198 (30.4)
Missing	3 (0.1)	3 (0.1)	6 (0.1)
Preterm birth			
No	6226 (94.7)	3546 (90.1)	9772 (93.0)
Yes	349 (5.3)	389 (9.9)	738 (7.0)
Postnatal psychological distress			
No	5563 (84.6)	3187 (81.0)	8750 (83.3)
Yes	717 (10.9)	658 (16.7)	1375 (13.1)
Missing	295 (4.5)	90 (2.3)	385 (3.6)
NICU Admission			
No	2232 (34.0)	1659 (41.9)	3891 (37.0)
Yes	429 (6.5)	471 (11.9)	899 (8.5)
Missing	3714 (59.5)	1826 (46.2)	5740 (54.5)

Data presented as n (%), except otherwise indicated. BMI, body mass index; NICU, neonatal intensive care unit; PPD, postnatal psychological distress. aComparison group reported no complications during pregnancy. bSee method for information on constructed variables

### Association between total number of pregnancy complications and depressive and anxiety disorders by 14 years postpartum

A total of 2760 (26.3%), 831 (7.9%), and 344 (3.3%) mothers reported one, two, and three or more complication(s) during pregnancy, respectively. The prevalence of depressive and anxiety disorders by 14 years postpartum among mothers who experienced one, two, three, or more pregnancy complications was 55.2%, 58.8%, and 66.3%, respectively, compared with 42.3% of mother who experienced no pregnancy complications. Compared to having no pregnancy complications, having at least one pregnancy complication was associated with increased odds of depressive and anxiety disorders by 14 years postpartum: fully adjusted odds ratio [(aOR), 1.54 (95% CI, 1.40–1.70)]. The fully aORs were 1.70 (95% CI, 1.46–1.99) for two pregnancy complications and aOR 2.11 (95% CI, 1.65–2.69) for ≥three pregnancy complications compared to no pregnancy complications (Model 4 in Table 2).

**Table 2:** Association between total number of pregnancy complications and depressive and anxiety disorders by 14 years postpartum

Total no of PC(s)	No. of exposed cases	Model 1 OR (95% CI)	Model 2 OR (95% CI)	Model 3 OR (95% CI)	Model 4 OR (95% CI)
No PC (6575)	2784 (42.3)	1.00 (reference)	1.00 (reference)	1.00 (reference)	1.00 (reference)
1 PC (2760)	1526 (55.3)	1.68 (1.54–1.84)	1.69 (1.54–1.86)	1.52 (1.38–1.66)	1.54 (1.40–1.70)
2 PCs (831)	489 (58.8)	1.95 (1.69–2.26)	1.90 (1.63–2.22)	1.73 (1.49–2.02)	1.70 (1.46–1.99)
3 + PCs (344)	228 (65.3)	2.68 (2.13–3.36)	2.67 (2.11–3.39)	2.05 (1.61–2.60)	2.11 (1.65–2.69)

OR: Odd ratio, 95% CI: 95% Confidence Interval, PCs: Pregnancy complications, BMI: Body Mass Index

Data for depression/severe anxiety are presented as n (%)

Model 1: Unadjusted, Model 2: Adjusted for maternal age, ethnicity, area deprivation level, maternal education, household income, Model 3: Adjusted for maternal BMI, maternal smoking, alcohol consumption in pregnancy, longstanding illness, parity, child sex. Model 4: Fully adjusted

### Association between specific pregnancy complications and depressive and anxiety disorders by 14 years postpartum

A total of 632 (5.9%), 412 (3.9%), 772 (7.3%), and 191 (1.8%) mothers reported threatened miscarriage, bleeding in later pregnancy, preeclampsia, and GDM during pregnancy, respectively. The prevalence of depressive and anxiety disorders by 14 years postpartum among mothers who had threatened miscarriage, bleeding in later pregnancy, preeclampsia, and GDM was 56.2%, 57.4%, 57.3%, and 50.5% respectively. Compared to no pregnancy complications, the fully aORs for depressive and anxiety disorders were 1.38 (95% CI, 1.16–1.63) for threatened miscarriage, 1.33 (95% CI, 1.08–1.65) for bleeding in later pregnancy, and 1.43 (95% CI, 1.22–1.67) for preeclampsia. There was no evidence of an association between GDM and depressive and anxiety disorders by 14 years postpartum, aOR: [1.01 (95% CI, 0.74–1.37)], (Table 3).

**Table 3:** Association between pregnancy complications and depressive and anxiety disorders by 14 years postpartum

Pregnancy complications	No. of exposed cases	Unadjusted Model		^b^Adjusted Model	
		OR (95% CI)	*p*-value	OR (95% CI)	*p*-value
Threatened miscarriage					
No (9887)	273 (2.6)	1.00 (reference)		1.00 (reference)	
Yes (623)	350 (56.2)	1.43 ( 1.21–1.68)	< 0.001	1.38 (1.16–1.63)	< 0.001
Bleeding in later pregnancy					
No (10,102)	174 (1.7)	1.00 (reference)		1.00 (reference)	
Yes (408)	234 (57.8)	1.49 (1.22–1.82)	< 0.001	1.33 (1.08–1.65)	0.008
Preeclampsia					
No (9738)	330 (3.1)	1.00 (reference)		1.00 (reference)	
Yes (772)	442 (57.2)	1.51 (1.30–1.74)	< 0.001	1.43 (1.22–1.67)	< 0.001
Gestational diabetes mellitus					
No (10,320)	95 (0.9)	1.00 (reference)		1.00 (reference)	
Yes (190)	96 (50.0)	1.09 (0.82–1.46)	0.546*	1.01 (0.74–1.37)	0.916*

OR: Odd ratio, 95% CI: 95% Confidence Interval, PC: Pregnancy complications, BMI: Body Mass Index

Data for depression/severe anxiety are presented as n (%). aUnadjusted, bAdjusted for maternal age, ethnicity, area deprivation level, maternal education, household income, maternal BMI, maternal smoking, alcohol consumption in pregnancy, longstanding illness, parity, child sex

*P−value >0.05

### Subgroup and sensitivity analyses

The results of the subgroup analysis and interaction terms are presented in Table 4. There was no evidence that the association between pregnancy complications and depressive and anxiety by 14 years postpartum was modified by child sex, parity and area deprivation, models 1–3. In models 4 and 5, subgroup analysis found evidence of interaction for the association between pregnancy complication and depressive and anxiety disorders by 14 years postpartum among mothers with longstanding illness [p for interaction = 0.001], and mothers from other ethnicity [p for interaction = 0.004] respectively. In model 6, the association between pregnancy complications and depressive and anxiety disorders diagnosis by 14 years postpartum was higher among mothers who experienced postnatal psychological distress, aOR: [7.54 (95% CI, 5.99–9.49)], however, the difference was not statistically significant (p for interaction = 0.693). In model 7, the odds ratio for mothers with pregnancy complications who had preterm birth increased to aOR: [2.03 (95% CI, 1.62–2.55)] compared to mothers without a preterm birth, aOR: [1.56 (95% CI, 1.43–1.71)]. In model 8, the aOR was almost unchanged for mothers with pregnancy complications whose infants were admitted to NICU, aOR: [1.71 (95% CI, 1.38–2.13), p interaction = 0.683]. In model 9, only mothers of children diagnosed with autism had significantly higher odds of depressive and anxiety disorders by 14 years postpartum. However, the interaction did not reach statistical significance. Model 10 explored whether the association between pregnancy complications and depressive and anxiety disorders by 14 years postpartum differed according to support-seeking behaviour and found no evidence of interaction (*p* = 0.785).

**Table 4:** Subgroup analyses and interaction terms of the association between pregnancy complications and depressive and anxiety disorders by 14 years postpartum

Subgroups models	^a^No. in exposed group	^b^aOR (95% CI)	Interaction *P*-value
Model 1: Subgroup analysis by child sex			
Pregnancy complications (male child)	2019	1.59 (1.41–1.79)	0.830
Pregnancy complications (female child)	1915	1.63 (1.44–1.84)	
Model 2: Subgroup analysis by parity			
Pregnancy complications (with primiparous mothers)	2058	1.73 (1.53–1.96)	0.837
Pregnancy complications (with multiparous mothers)	1877	1.60 (1.42–1.83)	
Model 3: Subgroup analysis by area deprivation			
Pregnancy complications (with mother in most deprived areas)	2346	1.87 (1.64–2.12)	0.131
Pregnancy complications (with mothers in least deprived areas)	1589	1.49 (1.31–1.70)	
^*^Model 4: Subgroup analysis by longstanding illness			
Pregnancy complications (mothers with longstanding illness)	1195	3.14 (2.74–3.61)	0.001
Pregnancy complications (mothers without longstanding illness)	2738	1.74 (1.58–1.92)	
*Model 5: Subgroup analysis by mother’s ethnicity			
Pregnancy complications (mothers – ‘other’ ethnicity)	530	2.19 (1.75–2.73)	0.004
Pregnancy complications (mothers – white ethnicity)	3400	1.53 (1.39–1.68)	
Model 6: Interaction with postnatal psychological distress (PPD)			
Pregnancy complications (mothers with PPD)	658	7.54 (5.99–9.49)	0.693
Pregnancy complications (mother without PPD)	3187	1.56 (1.41–1.71)	
^*^Model 7: Interaction with being born preterm			
Pregnancy complications (child born preterm)	389	2.03 (1.62–2.55)	0.026
Pregnancy complications (child born full-term)	3546	1.56 (1.43–1.71)	
Model 8: Interaction with infant admission NICU			
Pregnancy complications (infants admitted to NICU)	466	1.71 (1.38–2.13)	0.683
Pregnancy complications (infant not admitted NICU)	1650	1.61 (1.40–1.85)	
Model 9: Interaction with child’s neurodevelopmental disorders at age 7			
Pregnancy complications + ADHD in child at age 7	54	1.70 (0.98–2.97)	0.321
Pregnancy complications + autism in child at age 7	61	2.49 (1.44–4.31)	0.963
Pregnancy complication + ADHD and Autism at age 7	17	1.39 (0.52–3.73)	0.975
Model 10: Interaction with seeking support after birth			
Pregnancy complications (Mothers – did not seek support)	1389	1.60 (1.39–1.84)	0.785
Pregnancy complications (Mothers – sought for support)	2543	1.56 (1.40–1.74)	

OR: Odd ratio, 95% CI: 95% Confidence Interval, PC: PPD; postnatal psychological distress, NICU; neonatal intensive care unit, ADHD, attention deficit hyperactivity disorder.

aExposure = Pregnancy complications

bAdjusted for maternal age, ethnicity, area deprivation level, maternal education, household income, maternal BMI, maternal smoking, alcohol consumption in pregnancy, longstanding illness, parity, child sex.

*P-value <.05

Adjusted estimates were similar to the primary analysis when we examined mothers participated at 14 years postpartum only (Table S1). Exclusion of mothers who had postnatal psychological distress at 9 months led to a slight decrease in aOR: [1PC: 1.41 (95% CI, 1.26–1.58)], [2PCs: 1.37 (95% CI, 1.14–1.66)] and [3 + PCs: 1.51 (95% CI, 1.17–2.04)] (Table S2). Results of the analysis excluding mothers who reported a mental health disorder as a longstanding illness were consistent with our primary analyses (Table S3 and S4). Adjusted results were not materially different when we examined the association between total number of pregnancy complications and depressive and anxiety disorders by 9 months, 3, 5, 7, and 11 years postpartum (Table S5). Adjusted associations of total number of pregnancy complications and depressive and anxiety disorders by 14 years postpartum, based on having complete exposure, covariate and outcome data were similar to our main results (Table S6). Finally, we found no difference in the characteristics of mothers included at baseline and 14 years postpartum (Table S7).

## Discussion

In this study, we found that the association between pregnancy complications and depressive and anxiety disorders by 14 years postpartum was stronger for mothers with multiple pregnancy complications compared to no pregnancy complications. Our study revealed that threatened miscarriage, bleeding in later pregnancy, and preeclampsia increased the odds of depressive and anxiety disorders in the mother by 14 years postpartum. However, the association did not reach statistical significance for GDM. In addition, these associations were consistent in the crude and fully adjusted models, indicating that the associations were not due to confounding factors. Although, point estimates were larger among mothers with pregnancy complication who also experienced postnatal psychological distress at 9 months postpartum, the interaction was not statistically significant (p for interaction = 0.693).

The results from this study corroborate findings from studies that have examined the relationship between various pregnancy complications and depressive and anxiety disorders. In a systematic review, Delahaije and colleagues synthesised evidence on depression and anxiety following preeclampsia. The authors reported mixed evidence, however, nearly all included studies reported a positive relationship [35]. Gaugler-Senden et al. found that higher scores for both depressive and anxiety disorders were reported in preeclamptic women during a 7-year follow-up period [21]. Two other studies reported no difference in depressive disorder diagnosis between women with preeclampsia and healthy controls at 2 and 15 months postpartum, respectively [36, 37] . With respect to anxiety disorders, three studies showed no significant association between preeclampsia and anxiety disorders [16, 37, 38]. although one study observed a higher anxiety score in women with preeclampsia compared to women without preeclampsia in the first postpartum year [37].

The associations between threatened miscarriage, bleeding in later pregnancy, and depressive and anxiety disorders have not received as much attention. Previous research on the role of miscarriage in longer-term depressive and anxiety disorders has focused on miscarriage resulting from recurrent miscarriage and spontaneous/induced abortion [13, 14]. Studies reporting on the association between threatened miscarriage have focused on short-term depressive and anxiety disorders [24, 39, 40]. In a cross-sectional study to estimate the extent of depressive and anxiety symptoms in women facing threatened miscarriage, it was reported that threatened miscarriage was strongly associated with depressive and anxiety disorders in a single 7-day timeframe [24]. Mirtabar et al., recent study indicated a similar result, women with threatened miscarriage experienced 1.8 to 1.9 times higher odds of experiencing depressive and anxiety symptoms [39]. Although these findings are consistent with the findings in this current study, the lack of specified timing for when depression and anxiety were measured, highlights the need for a temporal analysis in future longitudinal studies. Additionally, these findings support the need for integrated postpartum care that includes both short and long-term monitoring for women who experience threatened miscarriage. As there is no comprehensive review or prospective cohort study on depressive and anxiety disorders in the longer term, comparing the findings of our study on threatened miscarriage and bleeding in later pregnancy with previous research is challenging.

Our findings of no association between GDM and depressive and anxiety disorders are comparable with previous evidence from three population-based cohort studies [19, 20, 41]. In contrast, a nationwide cohort study that explored the association between GDM and psychiatric disorders beyond the first year postpartum found significant associations between previous GDM and incident depressive and anxiety disorders, as well as an increase in antidepressant medication [18]. The discrepancies in the association between GDM and depressive and anxiety disorders among previous studies may be due to a lower incidence of GDM in studies, leading to insufficient power to detect an association [19, 41]. Hence, population-based studies with larger sample sizes are warranted. Other studies report varying definitions of depressive and anxiety disorders and differences in confounding adjustment strategies [18, 20].

Our study found that the association between pregnancy complications and depressive and anxiety disorders was higher in mothers who experienced psychological distress in the postnatal period. Sun et al., suggests that psychological stress related to childbirth may contribute to continued depression and anxiety disorders in later life [42]. In a posthoc sensitivity analysis, we explored whether the association between pregnancy complications and depressive and anxiety disorders by 14 years postpartum differed according to postnatal support-seeking behaviour by including an interaction term. The subgroup analysis found a slight reduction in the odds ratio for mothers who sought support compared to mothers who did not seek support. However, the interaction was not statistically significant (*p* = 0.785). Additionally, mechanisms linking adverse pregnancy outcomes and depressive and anxiety disorders could also explain these associations [43]. Although we cannot infer a causal mechanism undermining the association between pregnancy complications and long-term depressive and anxiety disorders, it could be a potential prognostic factor for long-term maternal mental health. However, due to a lack of consensus on the evidence on the association between these pregnancy complications and long-term depressive and anxiety disorders, clinicians and health professionals pay less attention to the monitoring and intervention of these complications [44]. Nevertheless, minimising and managing multiple pregnancy complications as well as prioritising early screening for mothers with postnatal psychological distress may contribute to mother’s long-term mental health outcomes.

### Strengths and limitations

Some study limitations should be noted. First, depressive and anxiety disorders were operationalised in the MCS as a composite variable. Therefore, it was impossible to conduct separate analyses for depressive disorders and anxiety disorders. Second, while depressive and anxiety disorders are often co-morbid and share similar symptoms, they are distinct clinical disorders [45]. Thus, our findings may overlap with other studies that focus on a single mental health disorder. Third, whilst we conducted a sensitivity analysis (Table S4) excluding mothers who reported a mental health disorder as a “longstanding illness”, we were unable to study depressive and anxiety disorders before pregnancy. As a result, we were unable to examine the relationship between prenatal depressive and anxiety disorders and pregnancy complications. Fourth, we acknowledge the possibility of residual confounding due to unmeasured factors such as family/pre-pregnancy psychiatric history that may potentially have adverse outcomes on pregnancy and depressive and/or anxiety disorders. Fifth, our study sample size reduced from 18,246 to 10,510 by 14 years postpartum, which may indicate selection bias. However, the baseline characteristics were similar between those retained and those lost to follow-up (Table S7). Sixth, data on pregnancy complications were retrospectively self-reported by mothers at 9 months postpartum, data on pregnancy complications may be subject to recall bias. However, maternal reporting of pregnancy complication in the MCS has previously been compared to hospital records, and was found to be highly reliable with an agreement of 94–98% [46]. In addition, depressive/anxiety disorders were reliant on the mother’s reporting and may have been subject to social desirability bias [47, 48], as depressive or anxiety disorders may have been viewed as undesirable by some mothers. Objective measures of psychosocial factors, such as emotional or social support were unavailable in the MCS and is a limitation of our study as these may confound the association between pregnancy complications and long-term depressive and anxiety disorders. Future studies should explore the influence of detailed measures of psychosocial factors, such as emotional or social support on the association between pregnancy complications and long-term mental health outcomes. Finally, our results may not be generalisable to populations different from our study participants, therefore, the interpretation of this study’s findings should be viewed in the context of the above limitation.

The strengths of this study lie primarily in its large sample size and prospective data collection. We accounted for an array of potential confounders, including maternal socioeconomic status, prepregnancy, and lifestyle factors. The availability of these relevant data allowed for multiple sensitivity analyses to address residual confounding. Interaction terms were included to estimate the combined interaction between pregnancy complications and potential modifying factors. We excluded mothers who reported existing mental health disorders during pregnancy. Thus, exploring only new diagnoses of depressive and anxiety disorders since birth [49]. Finally, physician-diagnosed depressive and anxiety disorders are clinically validated [50]. However, as the first wave (MCS1) was collected at 9 months postpartum, the possibility of recall bias cannot be ruled out.

## Conclusion

Our study indicates that mothers who reported more pregnancy complications were at increased risk of later depressive and anxiety disorders and therefore may need to be prioritised for perinatal mental health intervention. These findings highlight the significant mental health burden on mothers experiencing preeclampsia, threatened miscarriage, and bleeding in later pregnancy, and may have implications for maternity care and perinatal mental health practices. We recommend that midwives and other clinicians prioritise the timely identification of mothers with multiple pregnancy complications, as well as facilitate early referral pathways for depressive and anxiety disorders. In the meantime, continued efforts to prevent and manage multiple pregnancy complications may benefit mother’s long-term mental health.

## Supplementary Information

Below is the link to the electronic supplementary material.


Supplementary Material 1


## Data Availability

The University of London Centre for Longitudinal Studies owns the copyright for the Millennium Cohort Study (MCS) data used in this study. Access is obtained via [https://www.closer.ac.uk/study/millennium-cohort-study/](https:/www.closer.ac.uk/study/millennium-cohort-study. Data are available from the UK Data Service [https://beta.ukdataservice.ac.uk/datacatalogue/series/series](https:/beta.ukdataservice.ac.uk/datacatalogue/series/series? id=2000031). For this study, we made use of the first six surveys (accession SN; MCS1-6: 4683, 5350, 5795, 6411, 7464, 8156, 8682).
