# Supplementary material for: Association between maternal pregnancy complications and long-term depressive and anxiety disorders: evidence from the UK millennium cohort study
Source: Soc Psychiatry Psychiatr Epidemiol. 2026 Apr 29;61(8):1463–74. doi: 10.1007/s00127-026-03094-4 (PMC13521974; doi:10.1007/s00127-026-03094-4)
Supplement: Supplementary file 1 [file 127_2026_3094_MOESM1_ESM.docx]

### **Supplementary Information**

## **Association Between Maternal Pregnancy Complications and Long-term Depressive and Anxiety Disorders: Evidence from the UK Millennium Cohort Study.**

Elizabeth O. BODUNDE, MPH^1,2*,^ Fergus P. MCCARTHY, PhD^2,3^, Karen O’CONNOR, MD^4,5^, Karen MATVIENKO-SIKAR,PhD^1^, Ali S. KHASHAN, PhD^1,2^.

1 School of Public Health; University College Cork, Cork Ireland.

2 INFANT Research Centre, University College Cork, Cork Ireland.

3 Department of Obstetrics and Gynaecology, Cork University Maternity Hospital, Cork, Ireland.

4 RISE, Early Intervention in Psychosis Team, South Lee Mental Health Services, Cork, Ireland.

5 Department of Psychiatry and Neurobehavioral Science, University College Cork, Ireland

***Corresponding Author:** Elizabeth Bodunde, School of Public Health, Western Gateway Building, University College Cork, Cork Ireland.

Phone +353 21 4205596 Email: [Seyibeth@gmail.com](mailto:Seyibeth@gmail.com)

ORCID: 0000-0002-0543-8349

Appendix S1: STROBE Statement

Appendix S2a: Pregnancy complications

Appendix S2b: List of specific pregnancy complications

Appendix S3: Constructed variables used in analysis

Fig S1: Graphs of cumulative prevalence of depressive and anxiety disorders

Table S1: Association between total number of pregnancy complications and depressive and anxiety disorders

by 14 years follow up only.

Table S2: Association between total number of pregnancy complications and depressive and anxiety disorders by 14 years postpartum (excluding mothers who had postnatal psychological distress at 9 months).

Table S3: Association between total number of pregnancy complications and depressive and anxiety disorders by 14 years postpartum (excluding mothers with history of prepregnancy longstanding mental disorders).

Table S4: Association between pregnancy complications and depressive and anxiety disorders by 14 years postpartum (excluding mothers with history of prepregnancy longstanding mental disorders).

Table S5: Association between total number of pregnancy complications and depressive and anxiety disorders at 9m, 3, 5, 7, and 11 years postpartum

Table S6: Association between total number of pregnancy complications and depressive and anxiety disorders by 14 years postpartum (only complete covariates).

Table S7: Characteristics of study population at baseline and participants from MCS 1 and MCS 6 included in the analysis.

Table S8: Characteristics of participants according to the number of complications. (All variables were reported at 9 months postpartum).

Table S9: Characteristics of participants according to specific pregnancy complications. (all variables were reported at 9 months postpartum).

| **Appendix S1: STROBE Statement** | | | |
| --- | --- | --- | --- |
|  | Item No | Recommendation | Page No |
| **Title and abstract** | 1 | (*a*) Indicate the study’s design with a commonly used term in the title or the abstract | 1, 2 |
|  |  | (*b*) Provide in the abstract an informative and balanced summary of what was done and what was found | 2 |
| Introduction | | | |
| Background/rationale | 2 | Explain the scientific background and rationale for the investigation being reported | 4, 5 |
| Objectives | 3 | State specific objectives, including any prespecified hypotheses | 5 |
| Methods | | | |
| Study design | 4 | Present key elements of study design early in the paper | 5 |
| Setting | 5 | Describe the setting, locations, and relevant dates, including periods of recruitment, exposure, follow-up, and data collection | 5, 6 |
| Participants | 6 | (*a*) Give the eligibility criteria, and the sources and methods of selection of participants. Describe methods of follow-up | 5, 6, Figure 1 |
|  |  | (*b*) For matched studies, give matching criteria and number of exposed and unexposed | NA |
| Variables | 7 | Clearly define all outcomes, exposures, predictors, potential confounders, and effect modifiers. Give diagnostic criteria, if applicable | 6, 7 |
| Data sources/ measurement | 8 | For each variable of interest, give sources of data and details of methods of assessment (measurement). Describe comparability of assessment methods if there is more than one group | 5 – 7 |
| Bias | 9 | Describe any efforts to address potential sources of bias | 6, 7 |
| Study size | 10 | Explain how the study size was arrived at | 5, Figure 1 |
| Quantitative variables | 11 | Explain how quantitative variables were handled in the analyses. If applicable, describe which groupings were chosen and why | 5, 7 |
| Statistical methods | 12 | (*a*) Describe all statistical methods, including those used to control for confounding | 7, 8 |
|  |  | (*b*) Describe any methods used to examine subgroups and interactions | 7, 8 |
|  |  | (*c*) Explain how missing data were addressed | 8 |
|  |  | (*d*) If applicable, explain how loss to follow-up was addressed | 8 |
|  |  | (*e*) Describe any sensitivity analyses | 7 |
| Results | | |  |
| Participants | 13 | (a) Report numbers of individuals at each stage of study—e.g. numbers potentially eligible, examined for eligibility, confirmed eligible, included in the study, completing follow-up, and analysed | 8, Table 1 |
|  |  | (b) Give reasons for non-participation at each stage | Table 1 |
|  |  | (c) Consider use of a flow diagram | Figure 1 |
| Descriptive data | 14 | (a) Give characteristics of study participants (e.g. demographic, clinical, social) and information on exposures and potential confounders | 8, Table 1 |
|  |  | (b) Indicate number of participants with missing data for each variable of interest | 8, Table 1 |
|  |  | (c) Summarise follow-up time (e.g., average and total amount) | 8, Table 1 |
| Outcome data | 15 | Report numbers of outcome events or summary measures over time | 9, Table 2 |
| Main results | 16 | (*a*) Give unadjusted estimates and, if applicable, confounder-adjusted estimates and their precision (e.g., 95% confidence interval). Make clear which confounders were adjusted for and why they were included | 9,  Table 2, 3 |
|  |  | (*b*) Report category boundaries when continuous variables were categorized | Supplementary file, appendix. |
|  |  | (*c*) If relevant, consider translating estimates of relative risk into absolute risk for a meaningful time period | NA |
| Other analyses | 17 | Report other analyses done—e.g. analyses of subgroups and interactions, and sensitivity analyses | 9, 10 Supplementary file. |
| Discussion |  |  |  |
| Key results | 18 | Summarise key results with reference to study objectives | 10, 11 |
| Limitations | 19 | Discuss limitations of the study, considering sources of potential bias or imprecision. Discuss both direction and magnitude of any potential bias | 12 -14 |
| Interpretation | 20 | Give a cautious overall interpretation of results considering objectives, limitations, multiplicity of analyses, results from similar studies, and other relevant evidence | 12-14 |
| Generalisability | 21 | Discuss the generalisability (external validity) of the study results | 14 |
| Other information |  |  |  |
| Funding | 22 | Give the source of funding and the role of the funders for the present study and, if applicable, for the original study on which the present article is based | 15 |

**Reference:** Information on the STROBE Initiative is available at <http://www.strobe-statement.org>.

| Appendix S2a: Construction of exposure variables | | |
| --- | --- | --- |
| Measures | **Question asked** | **Analysis variables** |
| Pregnancy complications | Mothers were asked, “Did you have any illness or problems during pregnancy”? If mothers responded positively, they were asked, “What illnesses or problems did you have”?  The data available had already been coded into illness categories to any of the following 20 pregnancy complications.  bleeding in early pregnancy/threatened miscarriage, bleeding in later pregnancy, multiple births, persistent vomiting, raised blood pressure/preeclampsia, urinary infections, diabetes, too much fluid around baby, suspected slow growth, ‘other’ anaemia, ‘other’ blood group disorders, ‘other’ joint problems, ‘other’ gall bladder problem, ‘other’ asthma, ‘other’ mental illness, ‘other’ neurological problems, ‘other’ uterine labor pain, ‘other’ early rupture membrane, ‘other’ suspected fetal problem, ‘other’ accident/injury. | We calculated the total number of complications for each mother and categorised them into “no pregnancy complication(s)”,  “1 pregnancy complication”,  “2 pregnancy complications”,  and “3+ pregnancy complications. |

| **Appendix S2b: Specific pregnancy complications reported by mothers at 9 months postpartum** | | |
| --- | --- | --- |
| **Self-reported maternal pregnancy complication** | **n (%)** | **Depressive and anxiety disorders by 14 years postpartum, n (%)** |
| - Raised blood pressure/preeclampsia | 772 (7.4) | 442 (57.3) |
| - Threatened miscarriage | 623 (5.9) | 350 (56.2) |
| - Bleeding in later pregnancy | 418 (3.9) | 238 (58.3) |
| - Gestational diabetes mellitus | 190 (1.8) | 96 (50.5) |
| - Multiple births |  | |
| - Persistent vomiting | 613 (5.8) | 369 (60.2) |
| - Urinary infections | 717 (6.8) | 443 (61.7) |
| - Too much fluid around baby | 99 (0.9) | 61 (61.6) |
| - Suspected slow growth | 214 (2.0) | 129 (60.3) |
| - ‘Other’ anaemia | 253 (2.4) | 125 (49.4) |
| - ‘Other’ blood group disorders | 110 (1.1) | 66 (60.0) |
| - ‘Other’ joint problems | 331 (3.2) | 211 (63.7) |
| - ‘Other’ gall bladder problem | 52 (0.5) | 25 (48.0) |
| - ‘Other’ asthma | 71 (0.7) | 43 (60.5) |
| - ‘Other’ mental illness | Excluded………………………………………. | |
| - ‘Other’ neurological problems | 86 (0.8) | 55 (63.9) |
| - ‘Other’ uterine labor pain | 80 (0.8) | 55 (68.7) |
| - ‘Other’ early rupture membrane | 29 (0.3) | 18 (62.1) |
| - ‘Other’ suspected fetal problem | 83 (0.8) | 49 (59.0) |
| - ‘Other’ not specified | 878 (8.4) | 526 (60.0) |

| Appendix S3: Constructed covariate used in analysis | | | |
| --- | --- | --- | --- |
| Measures | **Time** | **Question asked** | **Analysis variables** |
| Maternal age | 9 months | Maternal age measured in years and referred to mother’s age at time of birth of cohort member. | - 14-19 - 20-29 - 30-39 - 40+ |
| Maternal prepregnancy BMI | 9 months | Maternal height and weight just before becoming pregnancy were self-reported; these were used to derive maternal BMI. | - Underweight (min/18.49) - Normal weight (18.5/24.9) - Overweight (25.0/29.9) - Obese (30.0/max) |
| Maternal education | 9 months | Mother’s highest academic qualification was categorised as higher degree, first degree, diploma in higher educations, A/AS/S levels, O level/GSCE grades A -C, GSCE D-G, and other academic qualifications. | - None - Less than O levels - levels - A levels - Diploma/Higher - Otherprofessional qualification |
| Maternal ethnicity | 9 months | Mothers were asked ‘Which of the following do you regard yourself belonging to: White, Chinese, Irish Traveller, Indian, Pakistani, Bangladeshi, black Caribbean, black African, black other, mixed ethnic group, any other ethnic group’. | - White - Non-white |
| Parity | 9 months | Parity was derived from number of cohort member’s siblings in the household. | - Multiparous - Primiparous |
| Area deprivation | 9 months | Deprivation decile scores were calculated from home postcodes using the 2004 overall indices of multiple deprivation (IMD). For subgroup analyses, a binary variable was created to represent children living in areas of highest deprivation (deciles 1–5) and lowest deprivation (deciles 6–10) | - Most deprived (1-2) - Second decile (3-4) - Third decile (5-6) - Fourth decile (7-8) - Least deprived (9-10) |
| Smoking during pregnancy | 9 months | Mothers were asked the number of cigarettes they smoked per day before pregnancy and the number smoked per day if they changed the amount smoked during pregnancy. | - Nonsmoker - Quit smoking - Smoked during pregnancy |
| Alcohol consumption in pregnancy | 9 months | Mothers were asked about their frequency of alcohol consumption during pregnancy. | - Yes - No |
| Postnatal psychological distress (PPD) | 9 months | PPD measured using the modified nine-item Rutter Malaise Inventory (RMI-9) with an overall RMI score ≥ 4, indicating a sign of psychological distress. | - Yes - No |
| Autism | 7 years | Mothers were asked if a doctor or health professional had ever told them that their child has autism. | - Yes - No |
| Attention-deficit hyperactivity disorder (ADHD) | 7 years | Mothers were asked if a doctor or health professional had ever told them that their child has ADHD. | - Yes - No |
| Preterm birth | 9 months | Preterm birth is calculated by the child’s gestation age in days and converted to ≥ 37 weeks gestation | - Yes - No |
| Neonatal Intensive Care Unit (NICU) Admission | 9 months | Mothers were asked if their child was admitted to a special baby unit after birth | - Yes - No |

**Figure S1: Graphs of cumulative prevalence of depressive and anxiety disorders.**

| Table S1: Association between total number of pregnancy complications and depressive and anxiety disorders  by 14 years postpartum (Follow up at 14 years postpartum only). | | | |
| --- | --- | --- | --- |
| Total no of PC(s) | **No. of exposed cases** | **^a^OR (95% CI)** | **^b^OR (95% CI)** |
| Depressive and anxiety disorders at 14 years postpartum only | | | |
| No PCs | 1446 | Reference | Reference |
| 1 PC | 828 | 1.51 (1.36-1.67) | 1.36 (1.22-1.52) |
| 2 PCs | 285 | 1.82 (1.55-2.13) | 1.54 (1.30-1.82) |
| 3+ PCs | 143 | 2.65 (2.10-3.35) | 2.04 (1.59-2.63) |
| OR: Odd ratio, 95% CI: 95% Confidence Interval, PCs: Pregnancy complications, BMI: Body Mass Index.  ^a^Unadjusted  ^b^Adjusted for maternal age, ethnicity, area deprivation, maternal education, household income, maternal BMI, maternal smoking, alcohol consumption in pregnancy, longstanding illness, parity, child sex. | | | |

| Table S2: Association between total number of pregnancy complications and depressive and anxiety disorders by 14 years postpartum (excluding mothers who had postnatal psychological distress at 9 months). | | | |
| --- | --- | --- | --- |
| Total no of PC(s) | **No. of exposed cases** | **^a^OR (95% CI)** | **^b^OR (95% CI)** |
| Depressive and anxiety disorders by 14 years postpartum | | | |
| No PCs | 2128 | Reference | Reference |
| 1 PC | 1123 | 1.60 (1.45-1.76) | 1.41 (1.26-1.58) |
| 2 PCs | 360 | 1.86 (1.59-2.19) | 1.37 (1.14-1.66) |
| 3+ PCs | 156 | 2.37 (1.84-3.06) | 1.51 (1.17-2.04) |
| OR: Odd ratio, 95% CI: 95% Confidence Interval, PCs: Pregnancy complications, BMI: Body Mass Index.  ^a^Unadjusted  ^b^Adjusted for maternal age, ethnicity, area deprivation, maternal education, household income, maternal BMI, maternal smoking, alcohol consumption in pregnancy, longstanding illness, parity, child sex. | | | |

| Table S3: Association between total number of pregnancy complications and depressive and anxiety disorders by 14 years postpartum (excluding mothers with longstanding mental health disorders). | | | |
| --- | --- | --- | --- |
| Total no of PC(s) | **No. of exposed cases** | **^a^OR (95% CI)** | **^b^OR (95% CI)** |
| Depressive and anxiety disorders by 14 years postpartum | | | |
| No PCs | 2679 | Reference | Reference |
| 1 PC | 1443 | 1.65 (1.51-1.81) | 1.55 (1.40-1.70) |
| 2 PCs | 457 | 1.89 (1.63-2.19) | 1.71 (1.46-2.00) |
| 3+ PCs | 214 | 2.60 (2.07-3.29) | 2.13 (1.67-2.72) |
| OR: Odd ratio, 95% CI: 95% Confidence Interval, PCs: Pregnancy complications, BMI: Body Mass Index.  ^a^Unadjusted  ^b^Adjusted for maternal age, ethnicity, area deprivation, maternal education, household income, maternal BMI, maternal smoking, alcohol consumption in pregnancy, longstanding illness, parity, child sex. | | | |

| Table S4: Association between pregnancy complications and depressive and anxiety disorders by 14 years postpartum (excluding mothers with longstanding mental health disorders). | | | | | |
| --- | --- | --- | --- | --- | --- |
| Pregnancy complications | **No. of exposed cases** | **Unadjusted Model** | | **^b^Adjusted Model** | |
|  |  | **OR (95% CI)** | **p-value** | **OR (95% CI)** | **p-value** |
| Threatened miscarriage | |  |  |  |  |
| No (9668) | 273 (2.9) | 1.00 (reference) |  | 1.00 (reference) |  |
| Yes (605) | 332 (54.9) | 1.42 (1.20-1.67) | <0.001 | 1.38 (1.16-1.63) | <0.001 |
| Bleeding in later pregnancy | |  | |  | |
| No (9875) | 174 (1.8) | 1.00 (reference) |  | 1.00 (reference) |  |
| Yes (398) | 224 (56.3) | 1.46 (1.22-1.83) | <0.001 | 1.39 (1.12-1.72) | 0.006 |
| Preeclampsia |  |  |  |  |  |
| No (9528) | 330 (3.5) | 1.00 (reference) |  | 1.00 (reference) |  |
| Yes (745) | 415 (55.7) | 1.48 (1.27-1.72) | <0.001 | 1.42 (1.21-1.67) | <0.001 |
| Gestational diabetes | |  | |  | |
| No (10,088) | 95 (0.9) | 1.00 (reference) |  | 1.00 (reference) |  |
| Yes (185) | 90 (48.7) | 1.08 (0.81-1.45) | 0.584* | 1.04 (0.76-1.41) | 0.806* |
| OR: Odd ratio, 95% CI: 95% Confidence Interval, PC: Pregnancy complications, BMI: Body Mass Index .  Data for depression/severe anxiety are presented as n (%). ^a^Unadjusted, ^b^Adjusted for maternal age, ethnicity, area deprivation, maternal education, household income, maternal BMI, maternal smoking, alcohol consumption in pregnancy, longstanding illness, parity, child sex.  *P-value >.05 | | | | | |

| Table S5: Association between total number of pregnancy complications and depressive and anxiety disorders at 9m, 3, 5, 7, and 11 years postpartum. | | | | | |
| --- | --- | --- | --- | --- | --- |
| Total no of PC(s) | **No. of exposed cases** | **Model 1 OR (95% CI)** | **Model 2 OR (95% CI)** | **Model 3 OR (95% CI)** | **Model 4 OR (95% CI)** |
| Depressive and anxiety disorders at 9 months postpartum | | | | | |
| No PCs | 1240 | Reference | Reference | Reference | Reference |
| 1 PC | 783 | 1.70 (1.53-1.89) | 1.69 (1.53-1.89) | 1.52 (1.36-1.69) | 1.52 (1.36-1.70) |
| 2 PCs | 280 | 2.19 (1.87-2.56) | 2.11 (1.79-2.47) | 1.92 (1.63-2.26) | 1.87 (1.58-2.21) |
| 3+ PCs | 145 | 3.13 (2.51-3.92) | 3.10 (2.47-3.90) | 2.39 (1.89-3.02) | 2.43 (1.91-3.07) |
| Depressive and anxiety disorders at 3 years postpartum | | | | | |
| No PCs | 1820 | Reference | Reference | Reference | Reference |
| 1 PC | 1056 | 1.62 (1.47-1.780 | 1.62 (1.47-1.78) | 1.46 (1.32-1.61) | 1.48 (1.34-1.63) |
| 2 PCs | 358 | 1.98 (1.71-2.30) | 1.92 (1.64-2.23) | 1.76 (1.51-2.05) | 1.72 (1.47-2.00) |
| 3+ PCs | 171 | 2.58 (2.08-3.21) | 2.57 (20.5-3.22) | 2.00 (1.59-2.51) | 2.05 (1.62-2.58) |
| Depressive and anxiety disorders at 5 years postpartum | | | | | |
| No PCs | 2098 | Reference | Reference | Reference | Reference |
| 1 PC | 1219 | 1.68 (1.54-1.84) | 1.70 (1.54-1.86) | 1.52 (1.39-1.67) | 1.55 (1.40-1.70) |
| 2 PCs | 406 | 2.04 (1.77-2.36) | 1.98 (1.71-2.31) | 1.82 (1.57-2.11) | 1.79 (1.53-2.08) |
| 3+ PCs | 188 | 2.57 (2.07-3.20) | 2.56 (2.04-3.20) | 1.98 (1.58-2.49) | 2.02 (1.61-2.55) |
| Depressive and anxiety disorders at 7 years postpartum | | | | | |
| No PCs | 2326 | Reference | Reference | Reference | Reference |
| 1 PC | 1312 | 1.65 (1.51-1.81) | 1.66 (1.51-1.82) | 1.50 (1.36-1.64) | 1.51 (1.38-1.67) |
| 2 PCs | 428 | 1.94 (1.68-2.24) | 1.88 (1.62-2.19) | 1.73 (1.49-2.00) | 1.69 (1.45-1.97) |
| 3+ PCs | 201 | 2.57 (20.6-3.20) | 2.55 (2.03-3.20) | 1.99 (1.58-2.50) | 2.03 (1.60-2.56) |
| Depressive and anxiety disorders at 11 years postpartum | | | | | |
| No PCs | 2592 | Reference | Reference | Reference | Reference |
| 1 PC | 1436 | 1.66 (1.52-1.82) | 1.67 (1.53-1.84) | 1.51 (1.37-1.65) | 1.53 (1.39-1.68) |
| 2 PCs | 461 | 1.91 (1.66-2.21) | 1.86 (1.60-2.16) | 1.71 (1.47-2.00) | 1.67 (1.43-1.95) |
| 3+ PCs | 214 | 2.53 (2.02-3.16) | 2.52 (2.00-3.17) | 1.95 (1.55-2.46) | 2.00 (1.58-2.54) |
| OR: Odd ratio, 95% CI: 95% Confidence Interval, PCs: Pregnancy complications, BMI: Body Mass Index. Model 1: Unadjusted, Model 2: Adjusted for maternal age, ethnicity, area deprivation, maternal education, household income, Model 3: Adjusted for maternal BMI, maternal smoking, alcohol consumption in pregnancy, longstanding illness, parity, child sex. Model 4: Fully adjusted. | | | | | |

| Table S6: Association between total number of pregnancy complications and depressive and anxiety disorders by 9m, 3, 5, 7, and 11 years postpartum (including only complete co-variates). | | | |
| --- | --- | --- | --- |
| Total no of PC(s) | **No. of exposed cases** | **^a^OR (95% CI)** | **^b^OR (95% CI)** |
| Depressive and anxiety disorders at 9 months postpartum | | | |
| No PCs | 1237 | Reference | Reference |
| 1 PC | 781 | 1.70 (1.53-1.89) | 1.51 (1.36-1.69) |
| 2 PCs | 276 | 2.15 (1.84-2.52) | 1.84 (1.56-2.17) |
| 3+ PCs | 145 | 3.12 (2.50-3.90) | 2.42 (1.91-3.01) |
| Depressive and anxiety disorders at 3 years postpartum | | | |
| No PCs | 1813 | Reference | Reference |
| 1 PC | 1054 | 1.62 (1.47-1.780 | 1.48 (1.38-1.63) |
| 2 PCs | 354 | 1.95 (1.68-2.26) | 1.70 (1.45-1.99) |
| 3+ PCs | 171 | 2.58 (2.07-3.20) | 2.05 (1.62-2.58) |
| Depressive and anxiety disorders at 5 years postpartum | | | |
| No PCs | 2090 | Reference | Reference |
| 1 PC | 1215 | 1.68 (1.54-1.84) | 1.55 (1.40-1.71) |
| 2 PCs | 402 | 2.01 (1.74-2.33) | 1.76 (1.51-2.05) |
| 3+ PCs | 188 | 2.56 (2.06-3.19) | 2.02 (1.60-2.55) |
| Depressive and anxiety disorders at 7 years postpartum | | | |
| No PCs | 2316 | Reference | Reference |
| 1 PC | 1308 | 1.65 (1.51-1.81) | 1.52 (1.38-1.67) |
| 2 PCs | 424 | 1.92 (1.66-2.22) | 1.67 (1.43-1.95) |
| 3+ PCs | 201 | 2.56 (2.05-3.19) | 2.02 (1.60-2.55) |
| Depressive and anxiety disorders at 11 years postpartum | | | |
| No PCs | 2581 | Reference | Reference |
| 1 PC | 1428 | 1.66 (1.52-1.82) | 1.53 (1.39-1.68) |
| 2 PCs | 457 | 1.89 (1.64-2.19) | 1.65 (1.42-1.93) |
| 3+ PCs | 214 | 2.52 (2.02-3.15) | 1.99 (1.58-2.53_) |
| Depressive and anxiety disorders at 14 years postpartum | | | |
| No PCs | 2769 | Reference | Reference |
| 1 PC | 1517 | 1.67 (1.53-1.83) | 1.54 (1.40-1.69) |
| 2 PCs | 485 | 1.93 (1.67-2.24) | 1.68 (1.44-1.97) |
| 3+ PCs | 228 | 2.67 (2.13-3.36) | 2.11 (1.65-2.68) |
| OR: Odd ratio, 95% CI: 95% Confidence Interval, PCs: Pregnancy complications, BMI: Body Mass Index.  ^a^Unadjusted  ^b^Adjusted for maternal age, ethnicity, area deprivation, maternal education, household income, maternal BMI, maternal smoking, alcohol consumption in pregnancy, longstanding illness, parity, child sex. | | | |

| Table S7: Characteristics of study population at baseline and participants from MCS 1 and MCS 6 included in the analysis | | | | | |
| --- | --- | --- | --- | --- | --- |
| ****Characteristics Total N (%)**** | **Baseline cohort (n=18246)** | | | **14 years postpartum follow up (n=10510)** | |
|  | **No pregnancy complication** | **Pregnancy**  **complication** | | **No pregnancy**  **complication** | **Pregnancy complication** |
| Maternal age |  | |  |  |  |
| ≤35 | 9600 (84.4) | | 5789 (84.5) | 5418 (82.4) | 3263 (82.9) |
| ≥35 | 1778 (15.6) | | 1064 (15.5) | 1157 (17.6) | 672 (17.1) |
| Maternal BMI |  | |  |  |  |
| Normal (18.5-24.9 kg/m^2^) | **6920 (60.8)** | | **3942 (57.5)** | 4072 (61.9) | 2248 (57.1) |
| Underweight (<18.5 kg/m^2^) | **1659 (14.60** | | **914 (13.3)** | 858 (13.1) | 468 (11.9) |
| Overweight (25-29.9 kg/m^2^) | **2002 (17.6)** | | **1350 (19.7)** | 1182 (18.0) | 816 (20.7) |
| Obese (30-39 kg/m^2^) | **797 (7.0)** | | **647 (9.4)** | 463 (7.0) | 403 (10.3) |
| Child sex |  | |  |  |  |
| **Female** | **5587 (49.1)** | | **2378 (47.8)** | 3266 (49.7) | **1915** (48.7) |
| **Male** | **5789 (50.9)** | | **3574 (52.2)** | 3308 (50.3) | **2019** (51.3) |
| Missing | **2** | | **1** | 1 | 1 |
| ^b^Ethnicity |  | |  |  |  |
| **White** | **9350 (82.2)** | | **5929 (86.5)** | 5417 (82.4) | **3400** (86.4) |
| **Non-white** | **2007 (17.6)** | | **915 (13.3)** | 1146 (17.4) | **530** (13.5) |
| **Missing** | **21 (0.2)** | | **9 (0.2)** | **12** (0.2) | 5 (0.1) |
| ^b^Education |  | |  |  |  |
| **None of these** | **2333 (20.5)** | | **1210 (17.7)** | **1094** (16.6) | **529** (13.4) |
| **> O level** | **1245 (10.9)** | | **708 (10.3)** | **634** (9.6) | **389** (9.9) |
| **O level** | **3645 (32.0)** | | **2447 (35.7)** | **2052** (31.2) | **1368** (34.8) |
| **A level** | **1048 (9.2)** | | **645 (9.4)** | **649** (9.9) | **403** (10.2) |
| **Diploma/Higher** | **2719 (23.9)** | | **1658 (24.2)** | **1943** (29.6) | **1137** (28.9) |
| **Other qualification** | **362 (3.2)** | | **166 (2.4)** | **195** (3.0) | **104** (2.6) |
| **Missing** | **26 (0.2)** | | **19 (0.3)** | **8** (0.1) | **6** (0.2) |
| ^b^Area Deprivation |  | |  |  |  |
| **Most deprived** | **3826 (33.6)** | | **2091 (30.5)** | **1955** (29.7) | **1091** (27.7) |
| **Second decile** | **2557 (22.5)** | | **1595 (23.3)** | **1435** (21.8) | **889** (22.6) |
| **Third decile** | **1874 (16.5)** | | **1155 (16.8)** | **1155** (17.6) | **689** (17.5) |
| **Fourth decile** | **1489 (13.1)** | | **972 (14.2)** | **938** (14.3) | **633** (16.1) |
| **Least deprived** | **1632 (14.3)** | | **1039 (15.2)** | **1092** (16.6) | **633** (16.1) |
| Longstanding illness |  | |  |  |  |
| **No** | **9628 (84.6)** | | **4756 (69.4)** | **5558** (84.5) | **2738** (69.6) |
| **Yes** | **1745 (15.3)** | | **2095 (30.6)** | **1014** (15.4) | **1195** (30.3) |
| **Missing** | **5** | | **2** | **3** (0.1) | **2** (0.1) |
| ^b^Parity |  | |  |  |  |
| **Multiparous** | **5721 (50.3)** | | **3225 (47.1)** | **3342** (50.8) | **1877** (47.7) |
| **Primiparous** | **5657 (49.7)** | | **3628 (52.9)** | **3233** (49.2) | **2058** (52.3) |
| Income |  | |  |  |  |
| **Lowest quintile** | **2904 (25.5)** | | **1663 (24.3)** | **1372** (20.9) | **76** (19.4) |
| **Second quintile** | **2554 (22.4)** | | **1536 (22.4)** | **1343** (20.4) | **830** (21.1) |
| **Third quintile** | **2093 (18.4)** | | **1350 (19.7)** | **1240** (18.9) | **800** (20.3) |
| **Fourth quintile** | **1927 (19.9)** | | **1238 (18.1)** | **1311** (19.9) | **814** (20.7) |
| **Highest quintile** | **1856 (16.3)** | | **1050 (15.3)** | **1292** (19.6) | **724** (18.4) |
| **Missing** | **44 (0.4)** | | **16 (0.2)** | **17** (0.3) | **4** (0.1) |
| ^b^Maternal smoking during pregnancy |  | |  |  |  |
| **Nonsmoking** | **8253 (72.5)** | | **4750 (69.3)** | **4981** (75.8) | **2857** (72.6) |
| **Quit smoking** | **1353 (11.9)** | | **967 (14.1)** | **707** (10.8) | **516** (13.1) |
| **Smoked during pregnancy** | **1772 (15.6)** | | **1136 (16.6)** | **887 (13.4)** | **562** (14.3) |
| ^b^Alcohol consumption in pregnancy |  | |  |  |  |
| **No** | **8056 (70.8)** | | **4923 (71.8)** | **4528** (68.9) | **2778** (70.6) |
| **Yes** | **3322 (29.2)** | | **1926 (28.1)** | **2044** (31.0) | **1154** (29.3) |
| **Missing** |  | | **4 (0.1)** | **3** (0.1) | **3** (0.1) |
| Preterm birth |  | |  |  |  |
| **No** | **10737 (94.4)** | | **6151 (89.8)** | **6226** (94.7) | **3546** (90.1) |
| **Yes** | **641 (5.6)** | | **702 (10.2)** | **349** (5.3) | **389** (9.9) |
| ^b^PPD |  | |  |  |  |
| **No** | **9484 (83.4)** | | **5422 (79.1)** | **5563** (84.6) | **3187** (82.9) |
| **Yes** | **1372 (12.1)** | | **1241 (18.1)** | **717** (10.9) | **658** (17.1) |
| **Missing** | **522 (4.5)** | | **190 (2.8)** | **295** (4.5) | **90** (2.3) |
| NICU Admission |  | |  |  |  |
| **No** | **3791 (33.3)** | | **2851 (41.6)** | **2232** (34.0) | **1650** (41.9) |
| **Yes** | **745 (6.6)** | | **858 (12.5)** | **429** (6.5) | **466** (11.9) |
| **Missing** | **6842 (60.1)** | | **3144 (45.9)** | **3714** (59.5) | **1819** (46.2) |
| Data presented as n (%). BMI, body mass index; NICU, neonatal intensive care unit; PPD, postnatal psychological distress.  ^a^Comparison group reported no complications during pregnancy. ^b^See method for constructed on derived variables. | | | | | |

| ****Table S8: Characteristics of participants according to the number of complications. (All variables were reported at 9 months postpartum)**** | | | | | | |
| --- | --- | --- | --- | --- | --- | --- |
| Characteristics | **Total study sample (N)** | **^a^No pregnancy complications** | **1 pregnancy complication** | **2 pregnancy complications** | **3+ pregnancy complications** | ***P*** |
| Total (%) | 10510 (100.0) | 6575 (62.5) | 2760 (26.3) | **831 (7.9)** | **344 (3.3)** |  |
| Maternal age, n (%) |  |  |  |  |  |  |
| Mean (SD) | 28.9 (5.7) | 28.9 (5.8) | 28.9 (5.7) | 28.9 (5.6) | 28.4 (5.7) |  |
| 14-19 | 664 (6.3) | 408 (6.2) | 185 (6.7) | 51 (6.1) | 20 (5.7) | 0.848 |
| 20-29 | 4681 (44.6) | 2910 (44.3) | 1223 (44.3) | 381 (45.7) | 167 (48.7) |  |
| 30-39 | 4918 (46.8) | 3094 (47.1) | 1291 (46.9) | 380 (45.8) | 153 (44.4) |  |
| 40+ | 245 (2.3) | 161 (2.4) | 61 (2.1) | 19 (2.4) | 4 (1.2) |  |
| Missing | 2 | 2 | - | - | - |  |
| ^b^Maternal BMI |  |  |  |  |  |  |
| Normal (18.5-24.9 kg/m2) | 6320 (60.2) | 4072 (61.9) | 1597 (57.9) | 465 (55.9) | 186 (54.2) | <0.001 |
| Underweight (<18.5 kg/m^2^) | 1326 (12.6) | 858 (13.1) | 340 (12.4) | 90 (11.1) | 38 (10.9) |  |
| Overweight (25-29.9 kg/m^2^) | 1996 (19.0) | 1182 (18.0) | 560 (20.2) | 175 (21.0) | 81 (23.8) |  |
| Obese (30-39 kg/m^2^) | 866 (8.2) | 462 (7.0) | 263 (9.5) | 101 (12.0) | 39 (11.1) |  |
| Child sex |  |  |  |  |  |  |
| **Female** | **5181 (49.3)** | **3266 (49.7)** | **1340 (48.5)** | **419 (50.4)** | **156 (45.0)** | **0.644** |
| **Male** | **5327 (50.7)** | **3308 (50.3)** | **1419 (51.5)** | **412 (49.6)** | **188 (55.0)** |  |
| Missing | 2 | 1 | 1 | - | - |  |
| ^b^Ethnicity |  |  |  |  |  |  |
| **White** | **8817 (83.9)** | **5417 (82.4)** | **2361 (85.6)** | **737 (88.5)** | **302 (88.0)** | **<0.001** |
| Non-white | **1676 (15.9)** | **1146 (17.4)** | **395 (14.3)** | **93 (11.4)** | **42 (12.0)** |  |
| **Missing** | **17 (0.2)** | **12 (0.2)** | **4 (0.1)** | **1 (0.1)** | - |  |
| ^b^Education |  |  |  |  |  |  |
| **None of these** | **1623 (15.4)** | **1094 (16.6)** | **389 (14.1)** | **104 (12.5)** | **36 (10.6)** | **<0.001** |
| **> O level** | **1023 (9.7)** | **634 (9.6)** | **251 (9.1)** | **96 (11.5)** | **42 (12.3)** |  |
| **O level** | **3420 (32.5)** | **2052 (31.2)** | **942 (34.2)** | **305 (36.5)** | **121 (35.2)** |  |
| **A level** | **1052 (10.0)** | **649 (9.9)** | **279 (10.0)** | **83 (10.1)** | **41 (11.8)** |  |
| **Diploma/Higher** | **3080 (29.3)** | **1943 (29.6)** | **827 (30.0)** | **214 (25.9)** | **96 (27.8)** |  |
| **Other qualification** | **298 (2.8)** | **195 (3.0)** | **67 (2.4)** | **28 (3.4)** | **8 (2.3)** |  |
| **Missing** | **14 (0.3)** | **8 (0.1)** | **5 (0.2)** | **1 (0.1)** | **-** |  |
| ^b^Area Deprivation |  |  |  |  |  |  |
| **Most deprived** | **3046 (29.0)** | **1960 (29.7)** | **784 (28.3)** | **219 (26.6)** | **88 (25.5)** | **0.125** |
| **Second decile** | **2324 (22.1)** | **1443 (21.9)** | **607 (22.0)** | **195 (23.4)** | **87 (25.5)** |  |
| **Third decile** | **1844 (17.6)** | **1158 (17.5)** | **499 (18.1)** | **134 (16.1)** | **56 (16.0)** |  |
| **Fourth decile** | **1571 (14.9)** | **941 (14.3)** | **432 (15.7)** | **139 (16.5)** | **62 (17.8)** |  |
| **Least deprived** | **1725 (16.4)** | **1097 (16.6)** | **438 (15.9)** | **144 (17.4)** | **51 (15.2)** |  |
| Longstanding Illness | | | | | | |
| **No** | **8296 (78.9)** | **5558 (84.5)** | **1993 (72.3)** | **557 (67.0)** | **188 (55.0)** | **<0.001** |
| **Yes** | **2209 (21.0)** | **1014 (15.4)** | **765 (27.6)** | **274 (33.0)** | **156 (45.0)** |  |
| **Missing** | **5 (0.1)** | **3 (0.1)** | **2 (0.1)** | **-** | **-** |  |
| ^b^Parity |  |  |  |  |  |  |
| **Multiparous** | **5219 (49.7)** | **3342 (50.8)** | **1352 (49.0)** | **393 (47.3)** | **132 (38.7)** | **<0.001** |
| **Primiparous** | **5291 (50.3)** | **3233 (49.2)** | **1408 (51.0)** | **448 (52.7)** | **212 (61.3)** |  |
| Income |  |  |  |  |  |  |
| **Lowest quintile** | **2135 (20.3)** | **1372 (20.9)** | **537 (19.4)** | **159 (19.1)** | **67 (19.5)** | **0.015** |
| **Second quintile** | **2173 (20.7)** | **1343 (20.4)** | **555 (20.2)** | **200 (24.2)** | **75 (21.8)** |  |
| **Third quintile** | **2040 (19.4)** | **1240 (18.9)** | **592 (21.4)** | **144 (17.2)** | **64 (18.6)** |  |
| **Fourth quintile** | **2125 (20.2)** | **1311 (19.9)** | **549 (19.9)** | **180 (21.6)** | **85 (24.6)** |  |
| **Highest quintile** | **2016 (19.2)** | **1292 (19.6)** | **524 (19.0)** | **147 (17.8)** | **53 (15.5)** |  |
| **Missing** | **21 (0.2)** | **17 (0.3)** | **3 (0.1)** | **1 (0.1)** | **-** |  |
| ^b^Maternal smoking during pregnancy | | | | | | |
| Nonsmoker | **7838 (74.6)** | **4981 (75.8)** | **1996 (72.4)** | **630 (75.7)** | **231 (67.3)** | **<0.001** |
| **Quit smoking** | **1223 (11.6)** | **707 (10.7)** | **371 (13.4)** | **93 (11.3)** | **52 (14.9)** |  |
| **Smoked during pregnancy** | **1449 (13.8)** | **887 (13.5)** | **393 (14.2)** | **18 (13.0)** | **61 (17.8)** |  |
| bAlcohol consumption in pregnancy | | | | | | |
| **No** | **7306 (69.5)** | **4528 (68.9)** | **1935 (70.0)** | **591 (70.8)** | **252 (73.6)** | **0.406** |
| **Yes** | **3198 (30.4)** | **2044 (31.0)** | **823 (29.9)** | **239 (29.1)** | **92 (26.4)** |  |
| **Missing** | **6 (0.1)** | **3 (0.1)** | **2 (0.1)** | **1 (0.1)** | **-** |  |
| ^b^PPD | | | | | | |
| **No** | **8750 (83.3)** | **5563 (84.6)** | **2253 (81.7)** | **672 (81.0)** | **262 (76.2)** | **<0.001** |
| **Yes** | **1375 (13.1)** | **717 (10.9)** | **432 (15.6)** | **148 (17.6)** | **78 (22.4)** |  |
| **Missing** | **385 (3.4)** | **295 (4.5)** | **75 (2.7)** | **11 (1.4)** | **4 (1.4)** |  |
| NICU Admission |  |  |  |  |  |  |
| **No** | **3882 (37.0)** | **2232 (34.0)** | **1098 (39.8)** | **389 (46.8)** | **163 (47.3)** | **<0.001** |
| **Yes** | **895 (8.5)** | **429 (6.5)** | **278 (10.1)** | **118 (14.4)** | **70 (20.3)** |  |
| **Missing** | **5733 (54.5)** | **3914 (59.5)** | **1384 (50.1)** | **324 (38.8)** | **111 (32.4)** |  |
| Data presented as n (%), except otherwise indicated. BMI, body mass index; NICU, neonatal intensive care unit; PPD, postnatal psychological distress. ^a^Comparison group reported no complications during pregnancy. ^b^See method for information on constructed variables. | | | | | | |

| Table S9: Characteristics of participants according to specific pregnancy complications. (All variables were reported at 9 months postpartum). | | | | | | | | | | | | |
| --- | --- | --- | --- | --- | --- | --- | --- | --- | --- | --- | --- | --- |
| Characteristics | **Total Study**  **Participants** | **Mothers without**  **pregnancy complication** | **Mothers with pregnancy complications** | | | | | | | | |  |
|  |  |  | **Threatened miscarriage** | | **Bleeding in late pregnancy** | **Gestational diabetes** | | **Preeclampsia** | | | **Other PCs** |  |
| Total N (%) | 10510 (100.0) | 6575 (62.4) | 418 (4.1) | | 326 (3.1) | 160 (1.5) | | 772 (7.3) | | | 2259 (21.6) | *P* |
| Maternal age, n % |  |  |  | |  |  | |  | | |  |  |
| 14-19 | 664 (6.3) | 408 (6.2) | 21 (4.9) | | 32 (9.8) | 2 (1.2) | | 50 (6.5) | | | 151 (6.7) | 0.021 |
| 20-29 | 4681 (44.5) | 2910 (44.3) | 172 (41.3) | | 150 (46.2) | 59 (37.3) | | 364 (47.2) | | | 1026 (45.2) |  |
| 30-39 | 4918 (46.8) | 3094 (47.0) | 214 (51.2) | | 141 (43.1) | 96 (59.6) | | 341 (44.1) | | | 1032 (45.8) |  |
| 40+ | 245 (2.4) | 161 (2.5) | 11 (2.6) | | 3 (0.9) | 3 (1.9) | | 17 (2.2) | | | 50 (2.3) |  |
| Missing | 2 | 2 | - | | - | - | | - | | | - |  |
| ^b^Maternal BMI, n (%) |  |  |  | |  |  | |  | | |  |  |
| Normal (18.5-24.9 kg/m2) | **6320 (60.1)** | **4072 (61.9)** | **280 (66.4)** | | **203 (62.4)** | **72 (45.3)** | | **363 (47.0)** | | | **1330 (58.9)** | **<0.001** |
| Underweight (<18.5 kg/m^2^) | **1326 (12.7)** | **858 (13.1)** | **40 (9.6)** | | **33 (10.1)** | **22 (13.7)** | | **81 (10.5)** | | | **292 (13.1)** |  |
| Overweight (25-29.9 kg/m^2^) | **1998 (19.0)** | **1182 (18.0)** | **71 (17.6)** | | **72 (22.0)** | **35 (21.7)** | | **199 (25.8)** | | | **439 (19.3)** |  |
| Obese (30-39 kg/m^2^) | **866 (8.2)** | **463 (7.0)** | **27 (6.4)** | | **18 (5.5)** | **31 (19.3)** | | **129 (16.7)** | | | **198 (8.7)** |  |
| Child sex |  |  |  | |  |  | |  | | |  |  |
| **Female** | **5181 (49.3)** | **3266 (49.7)** | **205 (49.1)** | | **160 (49.2)** | **82 (50.9)** | | **373 (48.3)** | | | **1095 (48.3)** | **0.228** |
| **Male** | **5327 (50.7)** | **3308 (50.3)** | **212 (50.7)** | | **166 (50.8)** | **78 (49.1)** | | **399 (51.7)** | | | **1164 (51.7)** |  |
| Missing | **2** | **1 (0.0)** | **1 (0.2)** | | **-** | **-** | | **-** | | | **-** |  |
| ^b^Ethnicity |  |  |  | |  |  | |  | | |  |  |
| **White** | **8817 (83.9)** | **5417 (82.4)** | **378 (90.4)** | | **297 (91.1)** | **118 (73.9)** | | **682 (88.3)** | | | **1925 (85.2)** | **<0.001** |
| **Non-white** | **1676 (15.9)** | **1146 (17.4)** | **40 (9.6)** | | **28 (8.6)** | **42 (26.1)** | | **89 (11.5)** | | | **331 (14.7)** |  |
| **Missing** | **17 (0.2)** | **12 (0.2)** | **-** | | **1 (0.3)** | **-** | | **1 (0.2)** | | | **3 (0.1)** |  |
| bEducation |  |  |  | |  |  | |  | | |  |  |
| **None of these** | **1623 (15.4)** | **1094 (16.6)** | **42 (10.3)** | | **39 (11.9)** | **27 (16.8)** | | **84 (10.9)** | | | **337 (14.9)** | **<0.001** |
| **> O level** | **1023 (9.7)** | **634 (9.6)** | **35 (8.5)** | | **35 (11.0)** | **13 (8.1)** | | **76 (9.8)** | | | **230 (10.1)** |  |
| **O level** | **3420 (32.6)** | **2025 (31.2)** | **141 (33.3)** | | **127 (38.8)** | **52 (32.9)** | | **267 (34.6)** | | | **781 (34.6)** |  |
| **A level** | **1052 (10.0)** | **649 (9.9)** | **52 (12.7)** | | **24 (7.3)** | **16 (9.9)** | | **89 (11.5)** | | | **222 (9.8)** |  |
| **Diploma/Higher** | **3080 (29.3)** | **1943 (29.6)** | **139 (33.1)** | | **94 (28.8)** | **51 (31.7)** | | **237 (30.7)** | | | **616 (27.4)** |  |
| **Other qualification** | **298 (2.9)** | **195 (3.0)** | **8 (1.9)** | | **7 (2.2)** | **1 (0.6)** | | **19 (2.5)** | | | **68 (3.0)** |  |
| **Missing** | **14 (0.1)** | **8 (0.1)** | **1 (0.2)** | | **-** | **-** | | **-** | | | **5 (0.2)** |  |
| ^b^Area Deprivation |  |  |  | |  |  | |  | | |  |  |
| **Most deprived** | **3046 (29.0)** | **1955 (29.7)** | **89 (21.6)** | | **98 (30.0)** | **45 (28.0)** | | **197 (25.5)** | | | **662 (29.3)** | **0.051** |
| **Second decile** | **2324 (22.1)** | **1435 (21.8)** | **99 (23.5)** | | **63 (19.3)** | **36 (23.0)** | | **175 (22.7)** | | | **516 (22.8)** |  |
| **Third decile** | **1844 (17.6)** | **1155 (17.6)** | **86 (20.4)** | | **55 (17.1)** | **34 (21.1)** | | **136 (17.6)** | | | **378 (16.8)** |  |
| **Fourth decile** | **1571 (14.9)** | **938 (14.3)** | **68 (16.0)** | | **56 (17.1)** | **24 (14.9)** | | **125 (16.2)** | | | **360 (15.8)** |  |
| **Least deprived** | **1725 (16.4)** | **1092 (16.6)** | **76 (18.5)** | | **54 (16.5)** | **21 (13.0)** | | **139 (18.0)** | | | **343 (15.3)** |  |
| Longstanding Illness |  |  |  | | | | | | | | |  |
| **No** | **8296 (78.9)** | **5558 (84.5)** | **311 (74.4)** | | **233 (71.6)** | **94 (59.0)** | | **541 (70.1)** | | | **1559 (69.0)** | **<0.001** |
| **Yes** | **2209 (21.0)** | **1014 (15.4)** | **107 (25.6)** | | **93 (28.4)** | **66 (41.0)** | | **231 (29.9)** | | | **698 (30.9)** |  |
| **Missing** | **5 (0.1)** | **3 (0.1)** | **-** | | **-** | **-** | | **-** | | | **2 (0.1)** |  |
| bParity |  |  |  | |  |  | |  | | |  |  |
| **Multiparous** | **5219 (50.0)** | **3342 (50.8)** | **178 (42.7)** | | **149 (45.9)** | **96 (60.3)** | | **289 (37.4)** | | | **1165 (51.6)** | **<0.001** |
| **Primiparous** | **5391 (50.0)** | **3233 (49.2)** | **240 (57.3)** | | **177 (54.1)** | **64 (39.7)** | | **483 (62.6)** | | | **1094 (48.4)** |  |
| Income |  |  |  | |  |  | |  | | |  |  |
| **Lowest quintile** | **2135 (20.3)** | **1372 (20.9)** | **59 (14.1)** | | **69 (21.2)** | **30 (18.6)** | | **122 (15.8)** | | | **483 (21.4)** | **<0.001** |
| **Second quintile** | **2173 (20.7)** | **1343 (20.5)** | **76 (18.0)** | | **75 (23.2)** | **33 (21.2)** | | **139 (18.0)** | | | **507 (22.4)** |  |
| **Third quintile** | **2040 (19.4)** | **1240 (18.9)** | **90 (21.6)** | | **59 (18.0)** | **34 (21.1)** | | **165 (21.4)** | | | **452 (19.9)** |  |
| **Fourth quintile** | **2125 (20.2)** | **1311 (19.9)** | **107 (25.6)** | | **68 (20.8)** | **33 (20.5)** | | **183 (23.7)** | | | **423 (18.8)** |  |
| **Highest quintile** | **2016 (19.2)** | **1292 (19.7)** | **84 (20.2)** | | **55 (16.8)** | **30 (18.6)** | | **163 (21.1)** | | | **392 (17.4)** |  |
| **Missing** | **21 (0.2)** | **17 (0.1)** | **2 (0.5)** | | **-** | **-** | | **-** | | | **2 (0.1)** |  |
| ^b^Maternal smoking during pregnancy |  |  |  | | | | | | | | |  |
| **Nonsmoker** | **7838 (74.6)** | **4981 (75.8)** | **317 (75.8)** | | **227 (69.7)** | **129 (80.7)** | | **594 (76.9)** | | | **1590 (70.4)** | **<0.001** |
| **Quit smoking** | **1223 (11.6)** | **707 (10.7)** | **58 (13.9)** | | **45 (13.8)** | **22 (113.7)** | | **94 (12.2)** | | | **297 (13.1)** |  |
| **Smoked during pregnancy** | **1449 (13.8)** | **887 (13.5)** | **43 (10.3)** | | **54 (16.5)** | **9 (5.6)** | | **84 (10.9)** | | | **372 (16.5)** |  |
| ^b^Alcohol consumption in pregnancy |  |  |  | | | | | | | | |  |
| **No** | **7306 (69.5)** | **4528 (68.8)** | **272 (65.3)** | | **221 (67.9)** | **122 (76.4)** | | **565 (73.2)** | | | **1598 (70.5)** | **0.049** |
| **Yes** | **3198 (30.4)** | **2044 (31.1)** | **146 (34.7)** | | **105 (32.1)** | **38 (23.6)** | | **207 (26.8)** | | | **658 (29.4)** |  |
| **Missing** | **6 (0.1)** | **3 (0.1)** | **-** | | **-** | **-** | | **-** | | | **3 (0.1)** |  |
| bPPD |  |  |  | | | | | | | | |  |
| **No** | **8750 (83.3)** | **5563 (84.6)** | **346 (83.1)** | | **261 (79.8)** | **139 (86.3)** | | **631 (81.7)** | | | **1810 (80.1)** | **<0.001** |
| **Yes** | **1375 (13.0)** | **717 (10.9)** | **68 (16.0)** | | **61 (18.7)** | **16 (10.0)** | | **128 (16.6)** | | | **385 (17.0)** |  |
| **Missing** | **385 (6.7)** | **295 (4.5)** | **4 (0.9)** | | **5 (1.5)** | **5 (3.7)** | | **13 (1.7)** | | | **64 (2.9)** |  |
| NICU Admission |  |  |  | |  |  | |  | | |  |  |
| **No** | **3882 (36.9)** | **2232 (34.0)** | **182 (43.2)** | | **135 (41.3)** | **52 (32.9)** | | **340 (44.0)** | | | **941 (41.7)** | **<0.001** |
| **Yes** | **895 (8.5)** | **429 (6.5)** | **50 (12.7)** | | **51 (15.6)** | **41 (25.5)** | | **134 (17.4)** | | | **188 (8.4)** |  |
| **Missing** | **5733 (54.5)** | **3914 (59.5)** | **184 (44.1)** | **140 (43.1)** | | | **67 (41.6)** | | **298 (38.6)** | **1130 (49.9)** | |  |
| Data presented as n (%), except otherwise indicated. BMI, body mass index; NICU, neonatal intensive care unit; PPD, postnatal psychological distress. ^a^Comparison group reported no complications during pregnancy. ^b^See method for information on constructed variables. | | | | | | | | | | | | |

***Posthoc analysis**

| Table S10a: Association between total number of pregnancy complications and depressive and anxiety disorders by 14 years postpartum (adjusting for support seeking behaviour) | | | |
| --- | --- | --- | --- |
| Total no of PC(s) | **No. of exposed cases** | **^a^OR (95% CI)** | **^b^OR (95% CI)** |
| Depressive and anxiety disorders by 14 years postpartum | | | |
| No PCs | 2784 (42.3) | 1.00 (reference) | Reference |
| 1 PC | 1526 (55.3) | 1.68 (1.54-1.84) | 1.51 (1.38-1.67) |
| 2 PCs | 489 (58.8) | 1.95 (1.69-2.26) | 1.65 (1.41-1.93) |
| 3+ PCs | 228 (65.3) | 2.68 (2.13-3.36) | 2.01 (1.58-2.57) |
| OR: Odd ratio, 95% CI: 95% Confidence Interval, PCs: Pregnancy complications, BMI: Body Mass Index.  ^a^Unadjusted  ^b^Adjusted for maternal age, ethnicity, area deprivation, maternal education, household income, maternal BMI, maternal smoking, alcohol consumption in pregnancy, longstanding illness, parity, child sex, seeking support. | | | |

| Table S10b: Association between pregnancy complications and depressive and anxiety disorders by 14 years postpartum (adjusting for support seeking behaviour) | | | | | |
| --- | --- | --- | --- | --- | --- |
| Pregnancy complications | **No. of exposed cases** | **Unadjusted Model** | | **^b^Adjusted Model** | |
|  |  | **OR (95% CI)** | **p-value** | **OR (95% CI)** | **p-value** |
| Threatened miscarriage |  |  |  |  |  |
| No (9887) | 273 (2.6) | 1.00 (reference) |  | 1.00 (reference) |  |
| Yes (623) | 350 (56.2) | 1.43 ( 1.21-1.68) | <0.001 | 1.35 (1.13-1.61) | 0.001 |
| Bleeding in later pregnancy | |  | |  | |
| No (10,102) | 174 (1.7) | 1.00 (reference) |  | 1.00 (reference) |  |
| Yes (408) | 234 (57.8) | 1.49 (1.22-1.82) | <0.001 | 1.29 (1.04-1.60) | 0.002 |
| Preeclampsia |  |  |  |  |  |
| No (9738) | 330 (3.1) | 1.00 (reference) |  | 1.00 (reference) |  |
| Yes (772) | 442 (57.2) | 1.51 (1.30-1.74) | <0.001 | 1.39 (1.19-1.63) | 0.002 |
| Gestational diabetes mellitus | |  | |  | |
| No (10,320) | 95 (0.9) | 1.00 (reference) |  | 1.00 (reference) |  |
| Yes (190) | 96 (50.0) | 1.09 (0.82-1.46) | 0.546* | 0.97 (0.72-1.32) | 0.865* |
| ^OR: Odd ratio, 95% CI: 95% Confidence Interval, PC: Pregnancy complications, BMI: Body Mass Index .^  ^Data for depression/severe anxiety are presented as n (%). aUnadjusted, bAdjusted for maternal age, ethnicity, area deprivation level, maternal education, household income, maternal BMI, maternal smoking, alcohol consumption in pregnancy, longstanding illness, parity, child sex.^  ^*P-value >.05^ | | | | | |
